# A Case of BRASH Syndrome in an Elderly Female With Acute Urinary Retention

**DOI:** 10.7759/cureus.36803

**Published:** 2023-03-28

**Authors:** Rabia Mahmood, Ayman Mohamed

**Affiliations:** 1 Internal Medicine, Ascension St. John Hospital, Detroit, USA

**Keywords:** acute on chronic renal failure, av node dysfunction, junctional bradycardia, severe hyperkalemia, brash syndrome

## Abstract

The BRASH syndrome is an uncommon but serious medical condition that is distinguished by a confluence of symptoms that include bradycardia, renal failure, AV node dysfunction, shock, and hyperkalemia. Bradycardia associated with BRASH syndrome is often refractory to conventional management guided by advanced cardiac life support (ACLS), therefore prompt and appropriate intervention can only be administered in the setting of early recognition. The management of BRASH syndrome in elderly patients can prove to be particularly challenging, primarily because of pre-existing comorbidities that place these patients at increased risk of complications. We present the case of an 82-year-old female who presented to the emergency department with acute urinary retention. Initial laboratory evaluation revealed severe hyperkalemia and acute kidney injury. Her EKG showed bradycardia with a junctional rhythm. Medication reconciliation revealed multiple potassium-sparing and AV nodal-blocking agents. The patient's presentation was consistent with the BRASH syndrome and the patient was treated with potassium lowering and vasoactive agents. Her bradycardia resolved upon treatment of hyperkalemia. Her admission was complicated by renal replacement therapy given the degree of renal dysfunction however the patient was ultimately discharged after renal function improved. Upon discharge, the suspected precipitants of BRASH syndrome including beta blockers, mineralocorticoid receptor antagonists, and angiotensin receptor antagonists were all discontinued.

## Introduction

The BRASH syndrome is a rare but potentially life-threatening medical condition marked by bradycardia, renal failure, AV node block, shock, and hyperkalemia. It is understood that the pathophysiology involves the effect of AV nodal blocking agents in combination with hyperkalemia to synergistically cause severe bradycardia [1]. This syndrome results from a vicious cycle where renal failure causes hyperkalemia and may also lead to the buildup of certain AV nodal blockers (i.e., beta-blockers, calcium channel blockers). The synergistic effect of AV node blockers and hyperkalemia causes bradycardia resulting in reduced cardiac output leading to renal hypoperfusion and thus worsening renal failure [2]. Bradycardia associated with BRASH syndrome is often refractory to conventional management guided by advanced cardiac life support (ACLS), therefore prompt and appropriate intervention can only be administered in the setting of early recognition [3]. The syndrome demands a high index of suspicion since early diagnosis and treatment are critical to prevent irreversible organ damage. In this case report, we describe an elderly patient with BRASH syndrome who initially presented with nonspecific symptoms and discuss the challenges encountered during the management of the condition, particularly in the elderly population. Through this case study, we aim to emphasize the significance of early recognition, prompt intervention, and appropriate management strategies in mitigating the devastating consequence of BRASH syndrome.

## Case presentation

An 82-year-old female with a past medical history significant for essential hypertension, hyperlipidemia, type 2 diabetes mellitus, chronic kidney disease stage IIIB, and nonobstructive coronary artery disease presented to the emergency department with a chief complaint of urinary retention. The patient reported that for the past 2 days, she had been unable to urinate. The patient reported her symptoms were associated with generalized weakness and fatigue over the past several days. She stated that over the past two days, she had significantly reduced her oral intake secondary to generalized weakness and malaise. The patient endorsed mild nausea but denied any vomiting. The patient stated she lived alone and was compliant with all her home medication including carvedilol 12.5 mg twice daily, spironolactone 25 mg daily, losartan 25 mg daily, and gabapentin 100 mg daily. The patient denied any fever, chills, lightheadedness, dizziness, loss of consciousness, chest pain palpitations, vomiting, diarrhea, constipation, or lower extremity swelling.

**Figure 1:**
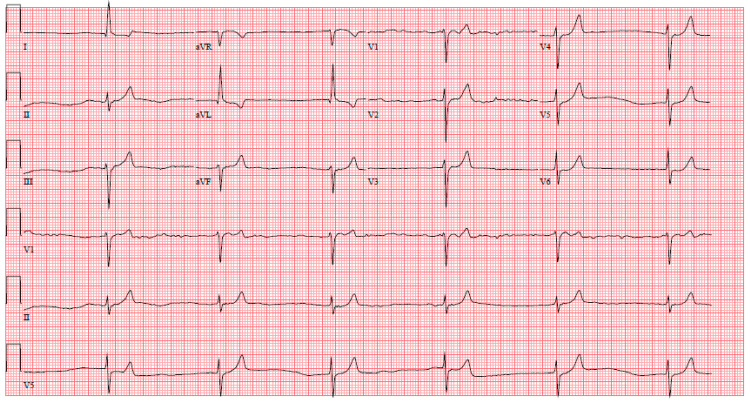
EKG on admission revealed sinus bradycardia with junctional rhythm, ventricular rate 37 bpm

On evaluation, the patient was overall alert and oriented, with intact cognitive function. She was cooperative with good judgment and insight. On vital examination the patient was afebrile 37.9 °C, however, was noted to be bradycardic with a ventricular rate of 37 beats/min and hypotensive with a blood pressure of 64/38 mm Hg. Physical examination was remarkable for dry mucous membranes and increased skin turgor. On abdominal examination, she was noted to have mild to moderate suprapubic tenderness to palpation and bladder distention. The remainder of her examination was within normal limits. The patient’s laboratory results were notable for creatinine elevated at 4.05 (from baseline 1.6 three months prior), blood urea nitrogen elevated at 50 mg/dL, and potassium elevated at 8.4 mEq/dL. Her anion gap was normal at 13 mmol/L and bicarbonate level was mildly reduced at 11 mmol/L. EKG revealed sinus bradycardia with a junctional rhythm, and heart rate at 37 bpm without interval changes or ST/T-wave changes (Figure 1).

The patient’s presentation and laboratory findings confirmed BRASH syndrome. The patient was promptly administered potassium lowering and vasoactive agents. She received 1 gram of IV calcium gluconate for cardiac membrane stabilization in the setting of severe hyperkalemia and bradycardia. She received nebulized albuterol, 10 units of insulin novolog with 1 ampule of 50% dextrose to shift potassium into the intracellular space. The patient was started on a dopamine drip to cause peripheral vaso-stimulation for hypotension. A foley catheter was inserted and maintained for acute urinary retention and revealed drainage of approximately 1,000 cc of urine from the urinary bladder. Repeat vitals after administration of IV calcium gluconate and potassium lowering agents revealed potassium improved at 6.6 mEq/dL. Repeat vitals revealed heart rate improved to 64 bpm. Blood pressure improved to 139/61 mm Hg and dopamine drip was gradually discontinued. In the ED vascular surgery was consulted for emergent femoral dialysis catheter placement. Nephrology was consulted for emergent dialysis. Cardiology was consulted for the evaluation of BRASH syndrome. During her admission the patient underwent one cycle renal replacement therapy. Her potassium normalized day 2 of admission to 4.6 mEq/dL. The patient’s creatinine downtrended to 2.74 mg/dL during her stay which was suspected to be multifactorial secondary to hemodynamic acute tubular necrosis with component of obstructive uropathy secondary to acute urinary retention and as well as prerenal acute kidney injury secondary to severe dehydration. The patient continued to have adequate urine output during her stay and was maintained on continuous IV fluid hydration for hypovolemia. She successfully completed a trial of void that resulted in discontinuation of the foley catheter. The patient did not have subsequent episodes of bradycardia during her admission. The patient’s medication list was thoroughly investigated and her nonselective beta blocker, mineralocorticoid receptor antagonist, and angiotensin receptor antagonist were discontinued as they were suspected to be precipitants of BRASH syndrome. The patient was started on chlorthalidone for hypertension with plan for close follow-up with nephrology and cardiology in the outpatient setting in 3-5 days after discharge. The patient was successfully discharged home on day 4 of admission.

## Discussion

The acronym BRASH stands for the clinical pentad commonly observed in this syndrome: bradycardia, renal failure, AV nodal blockade, shock, and hyperkalemia. It is understood that the pathophysiology involves the effect of AV nodal blocking agents in combination with hyperkalemia to synergistically cause severe bradycardia [1]. This syndrome results from a vicious cycle where renal failure causes hyperkalemia and may also lead to the buildup of certain AV nodal blockers (i.e beta blockers, calcium channel blockers). The synergistic effect of AV node blockers and hyperkalemia causes bradycardia resulting in reduced cardiac output leading to renal hypoperfusion and thus worsening renal failure [2].

It is crucial to distinguish BRASH syndrome from pure hyperkalemia and pure AV node blocker toxicity, as it represents as an overlap between the two. Although life-threatening arrhythmias can occur at any potassium level, arrhythmia associated with pure hyperkalemia typically manifests when potassium levels go beyond 6.5 mEq/L [3]. It is also important to note that when EKG abnormalities are disproportionate to the degree of hyperkalemia, other contributory factors should be considered. In the case of BRASH syndrome, bradycardia results from the combined effects of hyperkalemia and AV nodal blockade [3,4]. In addition to disproportionate bradycardia, BRASH syndrome often presents without other EKG findings consistent with hyperkalemia such as widened QRS complex or peaked T waves. Furthermore, unlike pure AV node blocker toxicity, patients with BRASH syndrome always exhibit hyperkalemia as it is the hallmark of this condition. Lastly, while pure AV blocker toxicity usually requires ingestion of high doses of medication, patients with BRASH syndrome generally do not exceed their prescribed doses [4].

BRASH syndrome is predominantly observed in elderly patients with underlying cardiac disease that is actively prescribed beta blockers and nondihydropyridine calcium channel blockers. Baseline renal dysfunction with a lack of renal reserve function is not uncommon in these patients. Triggers of BRASH syndrome include dehydration, adjustment or increase in antihypertensive medications such as ACE Inhibitors, ARBs, beta-blockers, or nondihydropyridine calcium channel blockers (i.e., verapamil and diltiazem), the introduction of potassium-sparing diuretics (i.e., spironolactone), or any precipitant of acute kidney injury [4,5].

Timely identification and management of BRASH syndrome are critical for preventing adverse outcomes. The immediate management of hyperkalemia is aimed at membrane stabilization, shifting of potassium to the intracellular space, and administration of potassium wasting diuretics for potassium excretion. This involves IV calcium administration to stabilize the myocardium as well as IV insulin and dextrose to shift potassium intracellularly. Beta receptor agonists such as nebulized salbutamol or albuterol can also be used to shift potassium intracellularly and improve bradycardia [6]. Volume status greatly varies among presenting patients. It is not uncommon for patients to exhibit significant hypovolemia, particularly in cases of severe dehydration. As such, the administration of fluid resuscitation is essential in these individuals. However, it is important to exercise caution in patients who suffer from BRASH syndrome, as they are at an elevated risk for volume overload secondary to anuric renal failure. In instances of anuric renal failure or refractory hyperkalemia, dialysis must be given due consideration [7]. The efficacy of the ACLS bradycardia algorithm is compromised in the context of BRASH syndrome, as it is often refractory to conventional interventions such as atropine and transcutaneous pacing yet demonstrates favorable outcomes with the administration of intravenous calcium. IV epinephrine can also be considered to increase heart rate and cardiac output. It is also efficacious in shifting potassium intracellularly. However, unwarranted adherence to the ACLS algorithm in such cases can potentially result in excessive utilization of transvenous pacing [6-8]. BRASH syndrome represents a metabolic/toxicological disturbance that is amenable to pharmacology/medical intervention rendering emergent transvenous pacemaker placement largely unnecessary [7,8].

## Conclusions

The BRASH syndrome is a complex and life-threatening condition that is challenging to diagnose due to the nonspecific nature of the presenting symptoms. The management of BRASH syndrome in elderly patients can be especially difficult due to age-related physiologic changes and chronic comorbidities that place these patients at increased risk for grave complications, including multi-organ failure and death. This case underscores the significance of early recognition and management of BRASH syndrome, especially in elderly patients. Reliable diagnostic criteria and optimal management strategies are yet to be established and further research is needed in this area to aid in prompt recognition and early intervention. In the meantime, healthcare providers must exercise caution when prescribing medications associated with the development of BRASH syndrome, especially in patients at elevated risk, and maintain a strong index of suspicion for BRASH syndrome in any patient that presents with unexplained symptoms.
